# Risk factors and trajectories for self-harm, neurodevelopmental disorders and mental health conditions in pupils in alternative education provision in Wales: population-based electronic cohort study

**DOI:** 10.1192/bjo.2025.10827

**Published:** 2025-09-11

**Authors:** Olivier Y. Rouquette, Marcos del Pozo Baños, Sze Chim Lee, Ann John

**Affiliations:** Swansea University Medical School, Swansea University, UK; GESIS – Leibniz Institute for the Social Sciences, Cologne, Germany

**Keywords:** Childhood experiences, self-harm, mental health disorders, education and training, electronic records

## Abstract

**Background:**

Pupils in alternative education provision, known as ‘Educated in Other Than At School’ (EOTAS) in Wales, UK, are among the most vulnerable learners and who, for reasons such as mental health or behavioural challenges, do not attend a mainstream or special school.

**Aims:**

We compared self-harm, neurodevelopmental disorders and mental health conditions between EOTAS pupils and controls with similar characteristics, before and after being in EOTAS provision.

**Method:**

This population-based electronic cohort study included pupils in Wales aged 7–18 years, from the academic years 2010–11 to 2018–19. We linked data from Education Wales to primary and secondary healthcare records within the Secure Anonymised Information Linkage (SAIL) Databank. Individuals included in the EOTAS data-set were identified as cases. Controls were pseudo-randomly selected based on equivalent age and academic year distribution.

**Results:**

This study included 8056 pupils in EOTAS and 224 247 controls. Higher levels of deprivation, childhood maltreatment, self-harm, neurodevelopmental disorders and mental health conditions before EOTAS entry were linked to higher odds of being in EOTAS. Pupils in EOTAS provision had increased incidence of self-harm, neurodevelopmental disorders and mental health conditions, from 1 year after entering EOTAS provision up to 24 years of age, than pupils with similar characteristics not in EOTAS provision.

**Conclusion:**

While EOTAS provision plays an important role, our findings indicate that it is not sufficient on its own to meet pupils’ social, emotional, behavioural and mental health needs. Additional support and better integration with health and social services are required.

School-based research has proliferated in recent years in response to the recognition that this is an important setting for the prevention, support, early intervention and management of self-harm and mental health conditions in children.^1–3^ Most evidence focuses on mainstream school settings, thereby including the majority of school-age children. However, not all pupils attend mainstream schools. In the UK, ‘Educated in Other Than At School’ (EOTAS) is an alternative education provision designed to meet the specific needs of pupils who, for whatever reason, cannot attend a mainstream or special school.^4^ This includes those excluded from school either as formal disciplinary exclusion or a placement as an alternative to exclusion. EOTAS may also be used for reasons of health and safety where pupils’ characteristics (e.g. mental health, behavioural difficulties) are not considered suitable for mainstream school education.^4^ Since 2009/10, the number of pupils in EOTAS provision has shown an upward trend. Notably, the rate per 1000 pupils has more than doubled, from 2.1 in 2009/10 to 4.9 in 2023/24, indicating a significant rise in EOTAS provision over the years.^4^ While pupils in EOTAS provision are probably among the most vulnerable learners, they are underrepresented in research evidence. Findings are inconsistent in the few studies exploring the lived experiences of pupils in EOTAS.^5–7^ Pupils report greater flexibility and inclusion, and that their needs were better supported, but also inappropriate curricula, uneven support and few opportunities for success and reintegration. Nonetheless, to the best of the authors’ knowledge, no previous studies have examined pupils’ trajectories for self-harm and mental health condition outcomes before and after being in EOTAS provision.

With the linkage between routinely collected primary and secondary healthcare data, and education data covering the population of Wales, this study aimed to investigate self-harm, neurodevelopmental disorders and mental health conditions: (a) before being in EOTAS provision, (b) after being in EOTAS provision and (c) to compare these between pupils in EOTAS provision and comparable children from the general population.

## Method

**Fig. 1:**
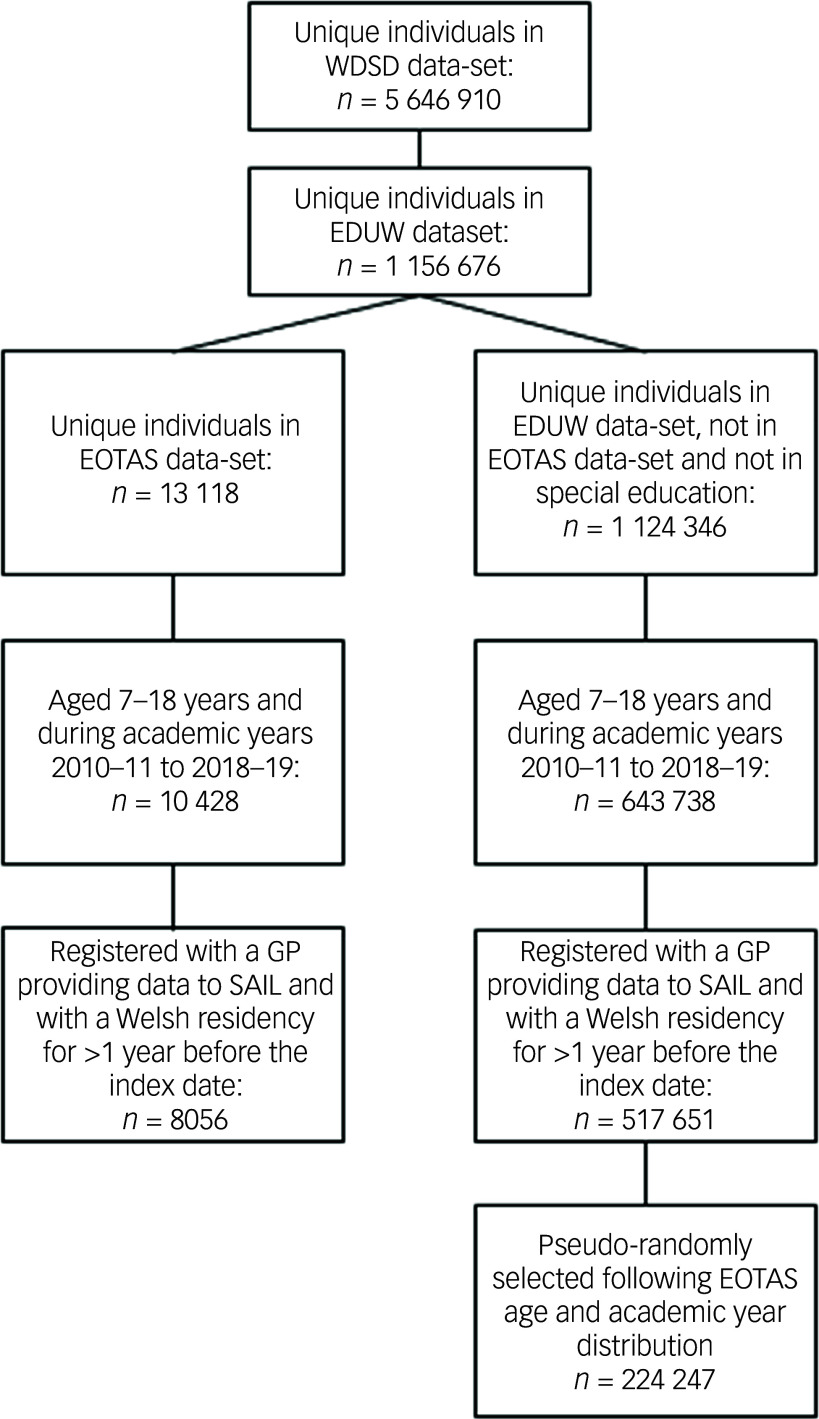
Flow diagram of cohort selection. WDSD, Welsh Demographic Service Dataset; EDUW, Education Wales; EOTAS, Educated in Other Than At School; GP, general practitioner; SAIL, Secure Anonymised Information Linkage.

This was a population-based electronic cohort study including data for pupils in Wales aged 7–18 years from the academic years 2010–11 to 2018–19 (Fig. 1). Ethical approval was granted by the Secure Anonymised Information Linkage (SAIL) Databank’s Independent Governance Review Panel (approval no. 0667).

### Data sources

We gathered information from Education Wales (EDUW) covering schools’ and pupils’ data for public-funded learning centres in Wales (including mainstream schools, special schools and EOTAS provision). The EDUW data-set contains information from the Pupil Level Annual School Census (PLASC) and the Welsh Examinations Database (WED). This describes learning centres, outcomes for learners, special educational needs (SEN), attendance summary (prior to 2020) and free school meals (FSM).^8^ We linked EDUW data from 2010–11 to 2018–19 within the SAIL Databank (www.saildatabank.com), a repository of routinely collected health and administrative data for the population of Wales, enabling individual-level linkage of data-sets without disclosing identifiable information.^9,10^ For EDUW and other data-sets used within SAIL, we used all records linked deterministically or probabilistically with a linkage score >0.9.^10^ In addition to the EDUW data, we used the following data-sets within SAIL for this study: Welsh Demographic Service data-set (a demographics register of people registered with a general practitioner (GP) in Wales); the Office for National Statistics deaths register; the Welsh Index of Multiple Deprivation 2011;^11^ the Welsh Longitudinal General Practice database (containing attendance and clinical information for all GP interactions and covering 86% of the population of Wales); and the Patient Episode database for Wales (hospital in-patient and day care activity in Wales).

### Cases – EOTAS pupils

Individuals included in the EOTAS data-set from EDUW, and aged 7–18 years from the academic years 2010–11 to 2018–19, were identified as cases. The index date was 1 September of the first academic year (i.e. 2010–11) in which they were recorded in an EOTAS provision. EOTAS data-sets were available for the academic year 2009–10, but individuals who registered for this academic year were censored due to a lack of certainty over whether it was their first entry in EOTAS provision.

### Controls

Individuals present in the EDUW data-set, aged 7–18 years between the academic years 2010–11 and 2018–19 and who were not in the EOTAS provision and not educated in a special school, were identified as controls. Pseudo-random index dates (start date) were generated following age distribution at the index date from EOTAS pupils per academic year and gender. Controls were subsequently pseudo-randomly selected following the distribution of year of birth from EOTAS cases. This ascertained a similar proportion of controls and cases per year of birth, and from similar age at entry, for each academic year.

### Inclusion criteria

The study period was between 1 January 2000 and 31 December 2019, using the SAIL Databank. Cases and controls were required to be registered with a GP practice in Wales for a minimum period of 1 year before the index date, and to have no conflicting data in the education data-set (e.g. one to many correspondences between the EDUW identifier and the anonymised linkage field in SAIL).

### Study population

For each individual, the index date was the first date of entry in EOTAS provision (for cases), or the pseudo-random index date (for controls). The follow-up period was from the most recent of either 1 January 2000, date of birth, start of registration with a GP providing data to SAIL or start of registration in a Welsh residency, to either the earliest date of end of registration in a Welsh residency, end of registration with a GP providing data to SAIL, death, 24 years of age (i.e. to allow for assessment of conditions more frequently diagnosed after school-leaving age with prodromal impacts during school)^12^ or end of the study period (31 December 2019).

### Measures and variables

#### EOTAS pupils

EOTAS status was included as a binary variable (0 for pupils not in EOTAS provision, 1 for pupils in EOTAS) and used as an outcome variable and a covariate or interaction, respectively, in analyses.

#### Self-harm, neurodevelopmental disorders and mental health conditions

During the follow-up time for each individual, we analysed primary care and hospital admission data-sets to identify self-harm and neurodevelopmental disorders – attention-deficit hyperactivity disorder (ADHD) and autism spectrum disorder (ASD) – and the following mental health conditions: learning difficulties, conduct disorder, depression, anxiety, eating disorder, alcohol misuse and drug misuse. This was done using validated Read code (version 2) lists and algorithms^13^ in primary care, and ICD-10 lists^14^ in secondary care from a previous study.^12^ Events of self-harm, neurodevelopmental disorders and mental health conditions were extracted using algorithms from the Adolescent Mental Health Data Platform (https://adolescentmentalhealth.uk/), with their definition, comprehensive code lists (Read codes and ICD-10 codes) and references openly accessible in the concept library (https://conceptlibrary.saildatabank.com/ADP/). Further details are available in Supplementary Table 1 available at https://doi.org/10.1192/bjo.2025.10827.

We created binary variables separately for self-harm, neurodevelopmental disorders and individual mental health conditions, to indicate the presence of the corresponding event any time before the index date (‘event before’). We identified new cases (incidences) of self-harm, neurodevelopmental disorders and individual mental health conditions from 1 year after the index date to the end of the observation period. We created a variable ‘morbidities’ by calculating the sum of first-time events of self-harm, neurodevelopmental disorders and mental health conditions for each individual during the follow-up period.

#### Covariates

Gender was entered as a binary variable (0 for female, 1 for male). Age at index date (first entry in EOTAS provision for EOTAS pupils, or pseudo-randomly generated index date for controls), in years, was computed as a continuous variable for each individual. We used the Welsh Index of Multiple Deprivation (an official measure of small area (defined as containing approximately 1500 individuals), based on employment opportunities, income, education, health, community safety, geographical access to services, housing and the physical environment) as a categorical variable, scoring from 1 (least deprived) to 5 (most deprived) based on quintiles. We also entered childhood maltreatment before the index date as a binary variable, due to the known consequences on pupils’ education outcomes.^15^ This was based on a validated algorithm using routinely collected data in primary and secondary care.^16^ SEN and FSM variables from the EDUW data-set were used in descriptive analyses, due only to their similarities with the deprivation quintile and events of self-harm, neurodevelopmental disorders and mental health, respectively.

### Analysis

We queried the data within the SAIL Databank using IBM DB2 9.7 SQL for Windows, and all statistical analyses were conducted in R-studio.^17–19^ The level of statistical significance was set at *P* = 0.05 with 95% confidence intervals. We summarised the characteristics of EOTAS pupils and controls using descriptive statistics (frequencies and percentages), with confidence intervals obtained by the Wilson score interval (WSI) with continuity correction,^20^ stratified by gender. We compared the age at entry in EOTAS for males and females using a Wilcoxon signed-ranked test with WSI continuity correction. We used logistic regression (with robust standard error to account for postential clustering in the data) to calculate the odds of being in EOTAS provision, stratified by gender and adjusting for deprivation quintile, child maltreatment, self-harm, neurodevelopmental disorders and mental health conditions occurring before the index date. We calculated unadjusted first-time incidence rate ratios (IRRs) of self-harm, neurodevelopmental disorders and mental health conditions from 1 year after the index date (first entry in EOTAS, or pseudo-random index date for controls, respectively) up to 24 years of age, with person-year at risk (PYAR) as the denominator. Subsequently, we used Poisson regressions (with robust standard errors) with PYAR as the denominator to calculate first-time IRRs of self-harm, neurodevelopmental disorders and mental health conditions from 1 year after the index date (first entry in EOTAS, or pseudo-random index date for controls, respectively) up to 24 years of age, adjusting for gender, deprivation quintile, child maltreatment, self-harm, neurodevelopmental disorders and mental health conditions before the index date. The underlying assumptions of Poisson regression, including overdispersion, goodness of fit, outliers, influential points and zero inflation, were carefully checked and met prior to conducting the analysis. We also computed Poisson regression models stratified by gender (available in Supplementary Tables 2 and 3), yielding similar results.

## Results

We included 8056 pupils in EOTAS provision and 224 247 controls aged 7–18 years between the academic years 2010–11 and 2018–19. Descriptive results (Table 1) show the differences in socioeconomic characteristics (i.e. deprivation quintiles), childhood maltreatment, self-harm, neurodevelopmental disorders and mental health conditions of pupils in EOTAS provision compared with the control group; 65.4% of pupils in EOTAS provision were male, versus 34.6% female. Compared with females, males in EOTAS provision were younger (mean age 13.2 years (s.d. = 2.2) versus 14.1 (s.d. = 1.5) for females, WSI = 8 951 566.5, *P* < 0.001); were from more deprived areas (with 40.4% of males versus 35.8% of females in EOTAS provision being from the most deprived areas in Wales); had higher rates of access to FSM (43.1 versus 39.7%, respectively); had a higher proportion of SEN compared with females (85.7 versus 71.2%, respectively); and more were statemented compared with females (19.6 and 9.6%, respectively). The prevalence of self-harm, neurodevelopmental disorders and mental health conditions before the index date also differed between males and females, particularly among EOTAS pupils.

**Table 1:** Demographic characteristics of control and EOTAS pupils

Characteristic	Control (*n* = 224 247)		EOTAS (*n* = 8056)	
	Female *n* (% [95% CI])	Male *n* (% [95% CI])	Female *n* (% [95% CI])	Male *n* (% [95% CI])
	111 083 (49.5 [49.3–49.7])	113 164 (50.5 [50.3–50.7])	2787 (34.6 [33.6–35.6])	5269 (65.4 [64.4–66.4])
Free school meals	17 995 (16.2 [16.0–16.4])	18 196 (16.1 [15.9–16.3])	1108 (39.7 [37.9–41.6])	2274 (43.1 [41.8–44.5])
Special educational needs (SEN)				
No SEN	92 031 (82.8 [82.6–83.0])	81 843 (72.3 [72.0–72.6])	803 (28.8 [27.1–30.5])	756 (14.3 [13.4–15.3])
Action	12 161 (10.9 [10.8–11.1])	17 696 (15.6 [15.4–15.8])	290 (10.4 [9.3–11.6])	482 (9.1 [8.4–9.9])
Action plus	5524 (5.0 [4.8–5.1])	9803 (8.7 [8.5–8.8])	1441 (51.7 [49.8–53.5])	3000 (57.0 [49.8–58.2])
Statemented	1270 (1.1 [1.1–1.2])	3738 (3.3 [3.2–3.4])	253 (9.1 [8.1–10.2])	1031 (19.6 [18.5–20.7])
Unknown	97	84		
Deprivation				
1 Least deprived	21 718 (19.5 [19.3–19.8])	22 786 (20.1 [19.9–20.4])	264 (9.5 [8.4–10.6])	403 (7.6 [6.9–8.4])
2	18 213 (16.4 [16.2–16.6])	18 811 (16.6 [16.4–16.8])	326 (11.7 [10.5–12.9])	538 (10.2 [9.4–11.0])
3	21 525 (19.4 [19.1–19.6])	21 885 (19.3 [19.1–19.6])	458 (16.4 [15.1–17.8])	820 (15.6 [14.6–16.5])
4	22 864 (20.5 [20.3–20.8])	23 338 (20.6 [20.4–20.8])	683 (24.5 [22.9–26.1])	1266 (24.0 [22.9–25.2])
5 More deprived	25 535 (23.0 [22.7–23.2])	25 235 (22.5 [22.3–22.8])	997 (35.8 [34.0–37.6])	2129 (40.4 [39.0–41.7])
Unknown	1228	1109	59	113
Events before index date (entry in EOTAS)				
Childhood maltreatment	4643 (4.0 [3.9–4.1)	4732 (4.2 [4.1–4.3])	620 (22.2 [20.7–23.8])	1163 (22.0 [20.1–23.2])
Self-harm	2069 (1.9 [1.8–1.9])	531 (0.5 [0.4–0.5])	491 (17.6 [16.2–19.0])	229 (4.3 [3.8–4.9])
ADHD	646 (0.6 [0.5–0.6])	2700 (2.4 [2.3–2.5])	148 (5.3 [4.5–6.2])	941 (17.8 [16.8–18.9])
ASD	375 (0.3 [0.3–0.4])	1757 (1.6 [1.5–1.6])	96 (3.4 [2.8–4.2])	373 (7.1 [6.4–7.8])
Learning difficulties	510 (0.5 [0.4–0.5])	1218 (1.1 [1.0–1.1])	73 (2.6 [2.1–3.3])	176 (3.3 [2.9–3.9])
Conduct disorder	690 (0.6 [0.6–0.7])	1444 (1.3 [1.2–1.3])	204 (7.3 [6.4–8.3])	514 (9.8 [9.0–10.6])
Depression	2648 (2.4 [2.3–2.5])	905 (0.8 [0.7–0.9])	434 (15.6 [14.3–17.0])	252 (4.8 [4.2–5.4])
Anxiety	3231 (2.9 [2.8–3.0])	1838 (1.6 [1.6–1.7])	413 (14.8 [13.6–16.2])	346 (6.6 [5.9–7.3])
Eating disorder	1271 (1.1 [1.1–1.2])	911 (0.8 [0.8–0.9])	96 (3.4 [2.8–4.2])	81 (1.5 [1.2–1.9])
Alcohol misuse	652 (0.6 [0.5–0.6])	346 (0.3 [0.3–0.3])	135 (4.8 [4.1–5.7])	103 (2.0 [1.6–2.4])
Drugs misuse	337 (0.3 [0.3–0.3])	212 (0.2 [0.2–0.2])	125 (4.5 [3.8–5.3])	106 (2.0 [1.7–2.4])
Morbidities (*n*)				
0	73 112 (65.8 [65.5–66.1])	86 106 (76.1 [75.8–76.3])	572 (20.5 [19.1–22.1])	1758 (33.3 [32.1–34.6])
1	23 305 (21.0 [20.7–21.2])	18 341 (16.2 [16.0–16.4])	663 (23.8 [22.2–25.4])	1474 (28.0 [26.8–29.2]
2	9717 (8.7 [8.6–8.9])	5764 (5.1 [5.0–5.2])	669 (24.0 [22.5–25.6])	983 (18.7 [17.6–19.7])
3	3239 (2.9 [2.8–3.0])	1925 (1.7 [1.6–1.8]	415 (14.9 [13.6–16.3])	546 (10.4 [9.6–11.2])
>3	1710 (1.5 [1.5–1.6])	1025 (0.9 [0.8–1.0])	468 (16.8 [15.4–18.2])	508 (9.6 [8.9–10.5])

EOTAS, Educated in Other Than At School; ADHD, attention-deficit hyperactivity disorder; ASD, autism spectrum disorder.

### Associations between history of self-harm, neurodevelopmental disorders and mental health conditions and being in EOTAS provision

**Fig. 2:**
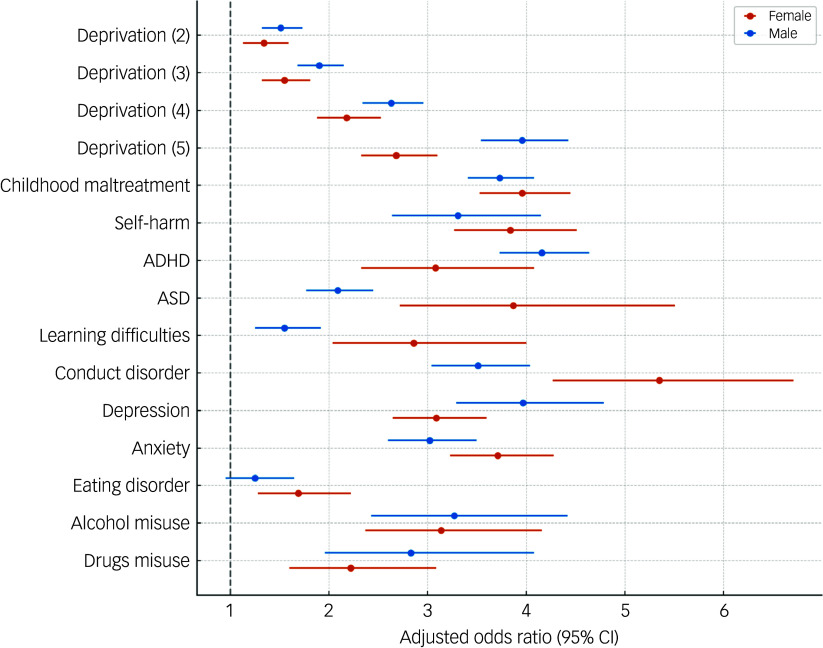
Adjusted odds ratios of being in Educated in Other Than At School provision, stratified by gender (female/male), from logistic regression with robust standard errors. Models were adjusted for deprivation (with (5) being the most deprived), childhood maltreatment, self-harm and neurodevelopmental and mental health conditions prior to the index date. ADHD, attention-deficit hyperactivity disorder; ASD, autism spectrum disorder.

We examined the associations between history of self-harm, neurodevelopmental disorders and mental health conditions and being in EOTAS provision (versus control) separately for males and females (Fig. 2). Overall, higher levels of deprivation, childhood maltreatment, self-harm, neurodevelopmental disorders and mental health conditions (except eating disorder for males) before the index date were linked with higher odds of being in EOTAS provision, for both genders. This was particularly the case for recorded diagnoses of conduct disorder in females (odds ratio 5.35) and ADHD in males (odds ratio 4.16) (see Supplementary Table 4 for full details).

### Incidence of self-harm, neurodevelopmental disorders and mental health conditions after being in EOTAS provision, compared with not in EOTAS provision

Unadjusted results showed higher incidences for all studied conditions from 1 year after being in EOTAS provision up to 24 years of age, compared with controls (Table 2).

**Table 2:** Crude incidence rate (95% CI) for self-harm, neurodevelopmental disorders and mental health conditions for controls and EOTAS pupils^a^

Events	Controls			EOTAS pupils		
	Count	PYAR	Incidence per 1000 PYAR (95% CI)	Count	PYAR	Incidence per 1000 PYAR (95% CI)
Self-harm	6579	1 141 919	5.76 (5.62–5.90)	817	38 056	21.47 (20.02–22.99)
ADHD	719	1 165 045	0.62 (0.57–0.66)	210	40 526	5.18 (4.50–5.93)
ASD	867	1 165 039	0.74 (070–0.80)	203	40 764	4.98 (4.32–5.71)
Learning difficulties	356	1 166 570	0.31 (0.27–0.34)	96	41 475	2.31 (1.87–2.83)
Conduct disorder	266	1 166 579	0.23 (0.20–0.26)	109	41 212	2.64 (2.17–3.19)
Depression	28 192	1 087 858	25.92 (25.61–26.22)	1798	35 648	50.44 (48.13–52.82)
Anxiety	20 414	1 112 368	18.35 (18.10–18.61)	1280	37 790	33.87 (32.04–35.78)
Eating disorder	1332	1 162 734	1.15 (1.08–1.21)	75	41 611	1.8 (1.42–2.26)
Alcohol misuse	3850	1 153 532	3.34 (3.23–3.44)	419	40 067	10.46 (9.48–11.51)
Drugs misuse	3480	1 157 461	3.01 (2.91–3.11)	734	38 838	18.9 (17.56–20.32)

EOTAS, Educated in Other Than At School; ADHD, attention-deficit hyperactivity disorder; ASD, autism spectrum disorder; PYAR, person-years at risk.

a From 1 year after entry in EOTAS (or pseudo-random index date for controls up to 24 years of age).

**Fig. 3:**
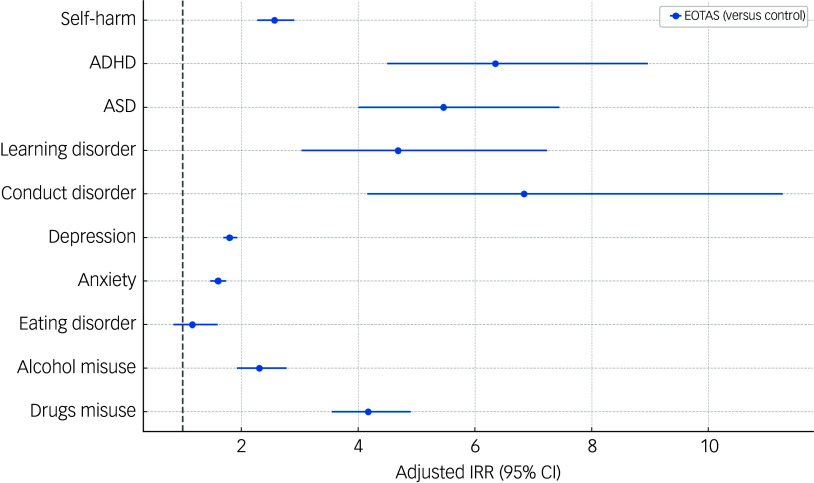
Incidence rate ratios (IRRs) for self-harm, neurodevelopmental disorders and mental health conditions from Poisson regression models, for pupils from 1 year after Educated in Other Than At School (EOTAS) (0/1) up to 24 years of age. Models were adjusted for EOTAS status (no/yes), gender (female/male) deprivation quintile, childhood maltreatment, self-harm, neurodevelopmental disorders and mental health conditions prior to index date. The full models are available in Supplementary Table 4. ADHD, attention-deficit hyperactivity disorder; ASD, autism spectrum disorder.

A summary of the results of Poisson regressions evaluating the first-time incidence of self-harm, neurodevelopmental disorders and mental health conditions from 1 year after the index date (first entry in EOTAS, or pseudo-random index date for EOTAS pupils and controls, respectively) up to 24 years of age can be seen in Fig. 3; the full results are available in Supplementary Table 4.

Pupils in EOTAS provision had a higher incidence of self-harm, neurodevelopmental disorders and mental health conditions (except eating disorder) from 1 year after the index date up to 24 years of age, compared with pupils with similar characteristics (i.e. gender, deprivation, childhood maltreatment, previous history of self-harm, neurodevelopmental disorders and mental health conditions) and who were not in EOTAS provision. The interaction between EOTAS (0/1) and gender (female:male) showed that males in EOTAS were at higher risk or self-harm, anxiety and eating disorder while females in EOTAS were at higher risk of ASD compared with their control counterparts (Supplementary Table 5). Findings from gender-stratified models were consistent with the main results, showing similar risk patterns across male and female pupils (see Supplementary Tables 2 and 3).

## Discussion

It is likely that pupils in EOTAS provision are among the most vulnerable learners, but there is scant research on their mental health needs. Our research allowed us to investigate self-harm, neurodevelopmental disorders and mental health conditions before and after being in EOTAS provision while accounting for gender, deprivation and childhood maltreatment, and to compare pupils in EOTAS provision with controls from general education of similar age and socioeconomic characteristics. Overall, higher levels of deprivation, childhood maltreatment, self-harm, neurodevelopmental disorders and mental health conditions before the index date were associated with higher odds of being in EOTAS provision. Pupils in EOTAS provision were more likely to be male, age at entry was younger for males and a higher proportion of females had comorbidities. Pupils in EOTAS provision had increased incidence of self-harm, neurodevelopmental disorders and mental health conditions (except eating disorder), from 1 year after entering EOTAS provision up to 24 years of age, than pupils with similar characteristics but not in EOTAS provision.

Overall, our results reveal that while females were fewer in number, they entered EOTAS provision at a later age compared with males and with more complex mental health needs (higher proportion of females with comorbidities). Similarly, and in line with the results of a recent study on the association between self-harm, neurodevelopmental disorders and mental health conditions and school absences and exclusions,^12^ our unadjusted results showed that pupils in EOTAS provision included a high proportion of females with events of self-harm, depression and anxiety before entry, while males in EOTAS provision had a higher proportion of ADHD, conduct disorder and ASD before entry. Furthermore, adjusted results showed that, despite lower proportions, females with an event of ASD, learning difficulties or conduct disorder had higher odds of being in EOTAS provision than males with similar events. This aligns with studies showing that neurodevelopmental disorders such as ASD and ADHD among females are generally diagnosed later compared with males, and with a higher proportion of females being first diagnosed with other conditions, including anxiety and depression, or potential misdiagnosis.^21–23^ This suggests that only the most severe cases were diagnosed among females, leading to an increased association of such cases with being in EOTAS provision, and with females in EOTAS provision having more severe and/or complex clinical needs requiring specific attention. Future research should explore the mechanisms underlying the observed gender differences in EOTAS entry, including potential diagnostic biases, differences in social support systems and variations in the presentation of neurodevelopmental and mental health conditions between males and females.

In line with the findings of a recent scoping review on alternative education settings,^24^ and with inspection reports of education and training in Wales on EOTAS,^25,26^ our results demonstrate that pupils in EOTAS provision were from significantly more deprived areas (particularly males) than their control counterparts. In parallel, a higher proportion of pupils in EOTAS had experienced childhood maltreatment. The association between deprivation level and childhood maltreatment is well established in the literature,^16,27^ as well as their consequences on pupils social, emotional and educational outcomes due to disruption of key developmental processes such as attachment relationships or emotion regulation.^15^

Our adjusted results subsequently showed that pupils in EOTAS provision had a higher incidence of self-harm, neurodevelopmental disorders and mental health conditions (except eating disorder) from 1 year after their entry in EOTAS provision up to 24 years of age, compared with pupils not in EOTAS but with similar characteristics. The increase in incidence in neurodevelopmental disorders, learning difficulties and conduct disorder may potentially reflect better access to services in those with EOTAS provision, allowing these conditions to be identified and assessed. However, it is worrying that our results demonstrate strong associations between being in EOTAS provision and subsequent risks of self-harm, depression, anxiety and alcohol and drugs misuse up to 24 years of age. This suggests the high level of need and complexity among children in EOTAS provision requiring increased support (e.g. social and emotional learning, skills-based learning) and integration with primary and secondary mental health services. These findings should also be considered in light of trauma-informed models. The elevated rates of diagnoses observed in this cohort probably reflect not the nature or effectiveness of EOTAS provision itself, but the cumulative adversity and complex needs of the young people accessing it. This perspective aligns with the trauma-informed approach embedded in Welsh education policy, which emphasises the importance of responding to pupils’ wider emotional and contextual experiences.

### Implications for policy and practices

An important proportion of pupils in EOTAS settings are from the most deprived areas in Wales, with complex life trajectories and social, emotional and mental health needs. Therefore, we recommend that EOTAS settings be better integrated and used as a bridge to inform, prepare and coordinate pupils’ transitions towards mental health services when required. Reflecting the proven usefulness of community services as an effective gateway,^28,29^ EOTAS provision is a unique opportunity to improve access, develop life skills and provide intensive support to the most vulnerable pupils. A key strategy that could improve outcomes for pupils in EOTAS is the whole-school approach (WSA), which focuses on creating an inclusive and supportive learning environment for all students. Evidence from Welsh schools shows that implementing explicit policies to remove learning barriers, appointing dedicated staff to monitor pupil progress and fostering a positive school culture can significantly improve engagement and reduce exclusions.^26^ By embedding these principles more widely, mainstream schools could better support at-risk students, potentially decreasing the number of pupils requiring EOTAS placements and improving long-term educational and social outcomes.

Given the levels of deprivation and maltreatment among pupils, it is important to equip educators and teachers in EOTAS provision settings with specialised training and knowledge to better understand and support pupils that have experienced childhood trauma.^15^ The implementation of a multi-level and comprehensive trauma-informed care (TIC) approach within organisations is vital.^30,31^ The TIC approach includes the need to realise and recognise that child maltreatment and adverse childhood events affect the health and development of individuals, and to respond by fully integrating knowledge about traumatic events into policy and practice and avoiding re-traumatisation.^30–32^ These findings are informing policy through ongoing engagement with the Welsh Government as part of the Administrative Data Research (ADR) Wales mental health programme, and are contributing to discussions on service planning and provision.

The findings also highlight the need for a more consistent approach to outcome monitoring across EOTAS settings. Consideration should be given to developing a standardised system for tracking educational and mental health outcomes, which would support clearer assessment of pupil trajectories and inform service planning across local authorities.

### Strengths and limitations

This study uniquely contributes to a better understanding of self-harm, neurodevelopmental disorders and mental health conditions before and after being in EOTAS provision, by linking education data with primary and secondary care, and socioeconomic data for the entire Welsh population over 9 academic years (2010–11 to 2018–19). Rather than using self- or proxy-reported measures that may be prone to bias in research on pupils, we used a validated code list to identify events of childhood maltreatment,^16^ self-harm, neurodevelopmental disorders and mental health conditions.^12^ However, the results should also be interpreted taking into account their limitations. A potential limitation of the study is that the inclusion criteria required cases and controls to be registered with a GP practice in Wales for at least 1 year prior to the index date. This may have led to an underestimation of the population, particularly for children from deprived backgrounds, who may not be registered with a GP. Although GP registration is generally automatic in the UK, this may still exclude vulnerable children who are not registered or may have limited access to healthcare.

We used routinely collected data that were not initially designed to be used in research, leading to potential misclassification, the presence of unmeasured confounding, missing data, having access to the sex but not the gender of individuals and poor recording of important variables such as ethnicity. These issues were addressed carefully, for instance by not including variables related to exclusions and absences in the analysis due to inconsistencies and lack of records in EOTAS provision.^33^ Because the study is based on observational data, it does not establish causal relationships between EOTAS provision and mental health outcomes. Other unmeasured factors may have contributed to the observed associations. Additionally, the study did not capture transitions out of EOTAS, such as re-integration into mainstream education or progression to further education, which remain important issues for separate examinination.

The variable for SEN from the education data-set showed a strong association with self-harm, neurodevelopmental disorders and mental health conditions from primary and secondary care, and was therefore excluded from analyses to avoid multicollinearity issues. Similarly, we excluded the variable for FSM from analyses because it strongly correlated with area deprivation; therefore, these two variables were used only in descriptive analysis. Bipolar disorder, schizophrenia and other psychotic disorders were not included in models due to low numbers, leading to lack of interpretability.

Dates available in the EOTAS data-set included only the academic year in which pupils were registered (but no specific date of entry or end date). Therefore, we took a conservative approach and set the date of entry as the first possible date (1 September) and the end date as the last possible date (31 August) for the academic year, leading to the establishment of a 1-year period before analysing events after the index date. This may have underestimated the association between being in EOTAS provision and self-harm, neurodevelopmental disorders and mental health conditions occurring after being in EOTAS provision. Future research should explore the causal mechanisms driving adverse trajectories in EOTAS pupils, as well as the long-term effectiveness of interventions aimed at supporting these pupils. Additionally, it is important to include underrepresented demographic variables, such as ethnicity and gender, to gain a more comprehensive understanding of the factors influencing outcomes in EOTAS settings.

This study showed that the majority of pupils in EOTAS provision have complex needs and are from the most deprived areas in Wales, with increased incidence of self-harm, neurodevelopmental disorders and mental health conditions from 1 year after EOTAS entry up to 24 years of age. EOTAS provision alone is not sufficient to address the social, emotional, behavioural and mental health needs of these pupils. Addressing the complex needs of this population requires the integration of EOTAS provision into health- and social care.

## Supporting information

Rouquette et al. supplementary material 1 Rouquette et al. supplementary material

Rouquette et al. supplementary material 2 Rouquette et al. supplementary material

Rouquette et al. supplementary material 3 Rouquette et al. supplementary material

Rouquette et al. supplementary material 4 Rouquette et al. supplementary material

## Data Availability

The data used in this study are available in the SAIL Databank at Swansea University, UK, via the Adolescent Mental Health Data Platform; however, because restrictions apply these are not publicly available. All proposals to use SAIL data are subject to review by an independent Information Governance Review Panel (IGRP). Before any data can be accessed, approval must be given by the IGRP. The IGRP gives careful consideration to each project to ensure proper and appropriate use of SAIL data. When access has been granted, it is gained through a privacy-protecting safe haven and remote access system referred to as the SAIL Gateway. SAIL has established an application process to be followed by anyone who wishes to access data via SAIL, at https://saildatabank.com/data/apply-to-work-with-the-data/.

## Supplementary material

The supplementary material is available online at https://doi.org/10.1192/bjo.2025.10827
