# Supplementary material for: Risk factors and trajectories for self-harm, neurodevelopmental disorders and mental health conditions in pupils in alternative education provision in Wales: population-based electronic cohort study
Source: BJPsych Open. 2025 Sep 11;11(5):e205. doi: 10.1192/bjo.2025.10827 (PMC12451533; doi:10.1192/bjo.2025.10827)
Supplement: Rouquette et al. supplementary material 2 — Rouquette et al. supplementary material [file S2056472425108272sup002.docx]

*Supplementary table 2 – (Female) Poisson model for self-harm, neurodevelopmental disorders and mental health conditions*.*

|  | **Self-harm** | | | **ADHD** | | | **ASD** | | | **Learning difficulties** | | | **Conduct disorder** | | |
| --- | --- | --- | --- | --- | --- | --- | --- | --- | --- | --- | --- | --- | --- | --- | --- |
| *Predictors* | *IRR* | *CI* | *p* | *IRR* | *CI* | *p* | *IRR* | *CI* | *p* | *IRR* | *CI* | *p* | *IRR* | *CI* | *p* |
| EOTAS [1] | 2.51 | 2.21 – 2.86 | <0.001 | 5.62 | 3.70 – 8.53 | <0.001 | 5.11 | 3.58 – 7.29 | <0.001 | 3.6 | 2.17 – 5.97 | <0.001 | 5.43 | 2.98 – 9.90 | <0.001 |
| Deprivation [2] | 1.11 | 0.99 – 1.24 | 0.065 | 0.73 | 0.46 – 1.16 | 0.188 | 1.28 | 0.86 – 1.90 | 0.226 | 1.69 | 0.95 – 3.03 | 0.076 | 1.08 | 0.49 – 2.41 | 0.845 |
| Deprivation [3] | 1.24 | 1.12 – 1.37 | <0.001 | 0.97 | 0.65 – 1.46 | 0.889 | 1.54 | 1.07 – 2.23 | 0.021 | 1.86 | 1.07 – 3.24 | 0.028 | 1.48 | 0.72 – 3.05 | 0.288 |
| Deprivation [4] | 1.4 | 1.27 – 1.54 | <0.001 | 1.24 | 0.85 – 1.81 | 0.258 | 1.5 | 1.05 – 2.16 | 0.027 | 1.99 | 1.16 – 3.42 | 0.013 | 1.5 | 0.74 – 3.05 | 0.265 |
| Deprivation [5] Most deprived | 1.51 | 1.37 – 1.66 | <0.001 | 0.93 | 0.63 – 1.37 | 0.716 | 1.36 | 0.95 – 1.95 | 0.093 | 2.12 | 1.25 – 3.62 | 0.006 | 2.3 | 1.19 – 4.45 | 0.013 |
| Childhood Maltreatment | 2.23 | 2.01 – 2.48 | <0.001 | 1.73 | 1.12 – 2.66 | 0.013 | 1.06 | 0.68 – 1.64 | 0.793 | 2.56 | 1.60 – 4.08 | <0.001 | 2 | 1.10 – 3.65 | 0.023 |
| Self-harm |  |  |  | 1.8 | 1.06 – 3.04 | 0.028 | 0.85 | 0.48 – 1.51 | 0.58 | 1.53 | 0.72 – 3.26 | 0.269 | 2.07 | 0.98 – 4.38 | 0.057 |
| ADHD | 1.5 | 1.15 – 1.95 | 0.003 |  |  |  | 5.51 | 3.33 – 9.12 | <0.001 | 6.11 | 3.17 – 11.80 | <0.001 | 4.35 | 1.79 – 10.58 | 0.001 |
| ASD | 0.9 | 0.58 – 1.37 | 0.614 | 2.22 | 0.85 – 5.82 | 0.104 |  |  |  | 2.97 | 1.16 – 7.62 | 0.023 | 0.74 | 0.11 – 5.00 | 0.753 |
| Learning difficulties | 1.19 | 0.85 – 1.68 | 0.304 | 4.36 | 2.25 – 8.43 | <0.001 | 4.74 | 2.72 – 8.25 | <0.001 |  |  |  | 1.42 | 0.33 – 6.13 | 0.634 |
| Conduct disorder | 1.68 | 1.32 – 2.14 | <0.001 | 2.05 | 1.02 – 4.12 | 0.043 | 1.98 | 1.10 – 3.54 | 0.022 | 1.73 | 0.75 – 3.98 | 0.196 |  |  |  |
| Depression | 2.42 | 2.09 – 2.79 | <0.001 | 2.11 | 1.30 – 3.43 | 0.003 | 2.65 | 1.73 – 4.08 | <0.001 | 0.67 | 0.26 – 1.74 | 0.409 | 1.87 | 0.90 – 3.88 | 0.091 |
| Anxiety | 1.35 | 1.16 – 1.58 | <0.001 | 1.4 | 0.83 – 2.36 | 0.201 | 2.33 | 1.59 – 3.43 | <0.001 | 0.99 | 0.45 – 2.13 | 0.97 | 1.1 | 0.44 – 2.74 | 0.839 |
| Eating disorder | 1.43 | 1.12 – 1.81 | 0.004 | 0.83 | 0.31 – 2.25 | 0.718 | 1.03 | 0.45 – 2.33 | 0.946 | 1.93 | 0.77 – 4.84 | 0.158 | 1.02 | 0.25 – 4.13 | 0.977 |
| Alcohol misuse | 2.39 | 1.89 – 3.02 | <0.001 | 1.01 | 0.39 – 2.65 | 0.978 | 0.56 | 0.18 – 1.75 | 0.321 | 0.67 | 0.15 – 3.01 | 0.606 | 1.25 | 0.42 – 3.66 | 0.689 |
| Drugs misuse | 2 | 1.31 – 3.05 | 0.001 | 0.21 | 0.03 – 1.45 | 0.114 | 0.84 | 0.25 – 2.82 | 0.776 | 0.85 | 0.19 – 3.87 | 0.831 | 2.17 | 0.80 – 5.87 | 0.128 |
| Observations | 112583 | | | 112583 | | | 112583 | | | 112583 | | | 112583 | | |
| R^2^ Nagelkerke | 0.041 | | | 0.056 | | | 0.07 | | | 0.059 | | | 0.07 | | |
|  |  |  |  |  |  |  |  |  |  |  |  |  |  |  |  |
|  | **Depression** | | | **Anxiety** | | | **Eating disorder** | | | **Alcohol misuse** | | | **Drugs misuse** | | |
| *Predictors* | *IRR* | *CI* | *p* | *IRR* | *CI* | *p* | *IRR* | *CI* | *p* | *IRR* | *CI* | *p* | *IRR* | *CI* | *p* |
| EOTAS [1] | 1.75 | 1.63 – 1.88 | <0.001 | 1.6 | 1.47 – 1.74 | <0.001 | 1.22 | 0.88 – 1.68 | 0.227 | 2.12 | 1.74 – 2.58 | <0.001 | 3.61 | 3.01 – 4.32 | <0.001 |
| Deprivation [2] | 1.07 | 1.02 – 1.13 | 0.006 | 1 | 0.94 – 1.05 | 0.917 | 0.92 | 0.76 – 1.10 | 0.339 | 1.09 | 0.93 – 1.28 | 0.281 | 1.04 | 0.86 – 1.26 | 0.689 |
| Deprivation [3] | 1.15 | 1.10 – 1.21 | <0.001 | 1.04 | 0.99 – 1.10 | 0.12 | 0.86 | 0.72 – 1.02 | 0.086 | 1.24 | 1.07 – 1.44 | 0.004 | 1.14 | 0.95 – 1.37 | 0.148 |
| Deprivation [4] | 1.32 | 1.27 – 1.39 | <0.001 | 1.15 | 1.09 – 1.21 | <0.001 | 0.71 | 0.59 – 0.85 | <0.001 | 1.2 | 1.04 – 1.40 | 0.013 | 1.5 | 1.27 – 1.78 | <0.001 |
| Deprivation [5] Most deprived | 1.44 | 1.38 – 1.51 | <0.001 | 1.16 | 1.10 – 1.22 | <0.001 | 0.71 | 0.59 – 0.84 | <0.001 | 1.31 | 1.14 – 1.51 | <0.001 | 1.56 | 1.32 – 1.83 | <0.001 |
| Childhood Maltreatment | 1.54 | 1.45 – 1.63 | <0.001 | 1.32 | 1.23 – 1.41 | <0.001 | 1.73 | 1.37 – 2.20 | <0.001 | 2.09 | 1.79 – 2.44 | <0.001 | 2.37 | 2.01 – 2.79 | <0.001 |
| Self-harm | 2.14 | 1.97 – 2.32 | <0.001 | 1.42 | 1.29 – 1.57 | <0.001 | 2.05 | 1.52 – 2.77 | <0.001 | 2.16 | 1.72 – 2.71 | <0.001 | 2.74 | 2.22 – 3.38 | <0.001 |
| ADHD | 1.17 | 1.01 – 1.34 | 0.033 | 1.46 | 1.25 – 1.69 | <0.001 | 1.37 | 0.78 – 2.40 | 0.273 | 1.77 | 1.26 – 2.49 | 0.001 | 1.74 | 1.23 – 2.45 | 0.002 |
| ASD | 1 | 0.80 – 1.25 | 0.982 | 1.35 | 1.07 – 1.70 | 0.011 | 1.11 | 0.48 – 2.58 | 0.805 | 0.6 | 0.28 – 1.28 | 0.185 | 0.41 | 0.17 – 0.98 | 0.045 |
| Learning difficulties | 1.02 | 0.85 – 1.23 | 0.824 | 0.9 | 0.72 – 1.13 | 0.371 | 1.17 | 0.58 – 2.38 | 0.659 | 0.96 | 0.56 – 1.66 | 0.888 | 0.72 | 0.40 – 1.31 | 0.285 |
| Conduct disorder | 1.57 | 1.39 – 1.77 | <0.001 | 1.29 | 1.11 – 1.51 | 0.001 | 0.97 | 0.53 – 1.77 | 0.922 | 1.28 | 0.89 – 1.83 | 0.181 | 1.7 | 1.24 – 2.33 | 0.001 |
| Depression |  |  |  | 2.04 | 1.88 – 2.21 | <0.001 | 1.54 | 1.13 – 2.08 | 0.006 | 1.69 | 1.36 – 2.11 | <0.001 | 2.05 | 1.65 – 2.55 | <0.001 |
| Anxiety | 1.61 | 1.49 – 1.72 | <0.001 |  |  |  | 1.69 | 1.29 – 2.22 | <0.001 | 0.98 | 0.76 – 1.27 | 0.898 | 1.01 | 0.78 – 1.30 | 0.932 |
| Eating disorder | 1.37 | 1.22 – 1.54 | <0.001 | 1.23 | 1.07 – 1.41 | 0.003 |  |  |  | 1.32 | 0.94 – 1.85 | 0.112 | 1.03 | 0.69 – 1.55 | 0.873 |
| Alcohol misuse | 1.37 | 1.20 – 1.56 | <0.001 | 1.27 | 1.09 – 1.48 | 0.002 | 1.13 | 0.66 – 1.95 | 0.652 |  |  |  | 2.57 | 1.89 – 3.49 | <0.001 |
| Drugs misuse | 1.14 | 0.95 – 1.37 | 0.163 | 0.94 | 0.76 – 1.16 | 0.565 | 0.51 | 0.21 – 1.26 | 0.143 | 1.53 | 0.99 – 2.37 | 0.056 |  |  |  |
| Observations | 112583 | | | 112583 | | | 112583 | | | 112583 | | | 112583 | | |
| R^2^ Nagelkerke | 0.049 | | | 0.034 | | | 0.012 | | | 0.027 | | | 0.071 | | |
| * Models adjusted for EOTAS status (No/Yes), deprivation quintile, childhood maltreatment, self-harm, ND, and mental health conditions before the index date. IRR = incidence rate ratio. | | | | | | | | | | | | | | | |

*Supplementary table 3 – (male) Poisson model for self-harm, neurodevelopmental disorders and mental health conditions*.*

|  | **Self-harm** | | | **ADHD** | | | **ASD** | | | **Learning difficulties** | | | **Conduct disorder** | | |
| --- | --- | --- | --- | --- | --- | --- | --- | --- | --- | --- | --- | --- | --- | --- | --- |
| *Predictors* | *IRR* | *CI* | *p* | *IRR* | *CI* | *p* | *IRR* | *CI* | *p* | *IRR* | *CI* | *p* | *IRR* | *CI* | *p* |
| EOTAS [1] | 3.26 | 2.90 – 3.66 | <0.001 | 6.73 | 5.41 – 8.37 | <0.001 | 3.53 | 2.77 – 4.49 | <0.001 | 4.36 | 2.97 – 6.41 | <0.001 | 7.42 | 5.28 – 10.43 | <0.001 |
| Deprivation [2] | 1.32 | 1.14 – 1.53 | <0.001 | 0.96 | 0.72 – 1.28 | 0.791 | 0.98 | 0.75 – 1.29 | 0.906 | 1.29 | 0.83 – 2.02 | 0.26 | 1.16 | 0.71 – 1.88 | 0.555 |
| Deprivation [3] | 1.35 | 1.18 – 1.55 | <0.001 | 1.09 | 0.84 – 1.43 | 0.519 | 1.07 | 0.83 – 1.38 | 0.613 | 1.09 | 0.70 – 1.70 | 0.706 | 1.33 | 0.84 – 2.10 | 0.226 |
| Deprivation [4] | 1.87 | 1.65 – 2.13 | <0.001 | 1.21 | 0.94 – 1.56 | 0.141 | 1.22 | 0.96 – 1.56 | 0.101 | 1.27 | 0.83 – 1.93 | 0.27 | 1.13 | 0.72 – 1.79 | 0.588 |
| Deprivation [5] Most deprived | 1.88 | 1.66 – 2.13 | <0.001 | 1.28 | 1.00 – 1.64 | 0.047 | 1.11 | 0.87 – 1.42 | 0.401 | 1.16 | 0.76 – 1.76 | 0.499 | 1.92 | 1.28 – 2.90 | 0.002 |
| Childhood Maltreatment | 1.78 | 1.56 – 2.02 | <0.001 | 1.93 | 1.49 – 2.51 | <0.001 | 1.42 | 1.08 – 1.87 | 0.011 | 1.83 | 1.23 – 2.72 | 0.003 | 1.69 | 1.12 – 2.54 | 0.012 |
| Self-harm |  |  |  | 0.98 | 0.49 – 1.96 | 0.962 | 0.43 | 0.15 – 1.19 | 0.104 | 0.41 | 0.11 – 1.56 | 0.189 | 1.07 | 0.42 – 2.73 | 0.886 |
| ADHD | 1.69 | 1.46 – 1.97 | <0.001 |  |  |  | 2.68 | 2.04 – 3.52 | <0.001 | 2.91 | 1.80 – 4.71 | <0.001 | 2.53 | 1.68 – 3.80 | <0.001 |
| ASD | 0.96 | 0.75 – 1.22 | 0.736 | 2.28 | 1.53 – 3.38 | <0.001 |  |  |  | 4.51 | 2.83 – 7.19 | <0.001 | 0.87 | 0.42 – 1.82 | 0.72 |
| Learning difficulties | 0.98 | 0.73 – 1.32 | 0.919 | 1.27 | 0.72 – 2.24 | 0.411 | 3.66 | 2.58 – 5.19 | <0.001 |  |  |  | 2.13 | 1.08 – 4.18 | 0.029 |
| Conduct disorder | 1.39 | 1.15 – 1.69 | 0.001 | 1.72 | 1.15 – 2.55 | 0.008 | 2.07 | 1.49 – 2.88 | <0.001 | 1.67 | 0.99 – 2.82 | 0.056 |  |  |  |
| Depression | 2.26 | 1.76 – 2.92 | <0.001 | 1.08 | 0.59 – 1.98 | 0.793 | 2.38 | 1.51 – 3.76 | <0.001 | 0.75 | 0.23 – 2.52 | 0.647 | 0.93 | 0.34 – 2.55 | 0.888 |
| Anxiety | 1.25 | 1.00 – 1.58 | 0.054 | 1.1 | 0.69 – 1.76 | 0.683 | 2.94 | 2.14 – 4.06 | <0.001 | 1.01 | 0.48 – 2.10 | 0.982 | 1.64 | 0.88 – 3.07 | 0.121 |
| Eating disorder | 1.22 | 0.86 – 1.74 | 0.259 | 0.94 | 0.42 – 2.13 | 0.89 | 2.1 | 1.23 – 3.60 | 0.007 | 1.45 | 0.53 – 3.99 | 0.467 | 1.09 | 0.35 – 3.44 | 0.88 |
| Alcohol misuse | 3.03 | 2.21 – 4.15 | <0.001 | 1.57 | 0.77 – 3.19 | 0.212 | 0.21 | 0.03 – 1.58 | 0.131 | 0.56 | 0.10 – 3.24 | 0.519 | 1.54 | 0.51 – 4.71 | 0.446 |
| Drugs misuse | 2.02 | 1.30 – 3.13 | 0.002 | 1.12 | 0.46 – 2.70 | 0.806 | 0.67 | 0.18 – 2.47 | 0.551 | 0.68 | 0.12 – 3.87 | 0.665 | 0.94 | 0.28 – 3.08 | 0.913 |
| Observations | 117211 | | | 117211 | | | 117211 | | | 117211 | | | 117211 | | |
| R^2^ Nagelkerke | 0.055 | | | 0.064 | | | 0.062 | | | 0.08 | | | 0.082 | | |
|  |  |  |  |  |  |  |  |  |  |  |  |  |  |  |  |
|  | **Depression** | | | **Anxiety** | | | **Eating disorder** | | | **Alcohol misuse** | | | **Drugs misuse** | | |
| *Predictors* | *IRR* | *CI* | *p* | *IRR* | *CI* | *p* | *IRR* | *CI* | *p* | *IRR* | *CI* | *p* | *IRR* | *CI* | *p* |
| EOTAS [1] | 1.82 | 1.69 – 1.95 | <0.001 | 1.98 | 1.81 – 2.16 | <0.001 | 1.73 | 1.07 – 2.82 | 0.026 | 2.45 | 2.12 – 2.85 | <0.001 | 4.33 | 3.84 – 4.88 | <0.001 |
| Deprivation [2] | 1.09 | 1.02 – 1.17 | 0.008 | 0.99 | 0.91 – 1.07 | 0.757 | 1.28 | 0.81 – 2.03 | 0.289 | 1.15 | 0.99 – 1.34 | 0.072 | 1.16 | 1.00 – 1.34 | 0.055 |
| Deprivation [3] | 1.15 | 1.09 – 1.23 | <0.001 | 1.06 | 0.98 – 1.14 | 0.155 | 1.31 | 0.84 – 2.03 | 0.229 | 1.31 | 1.14 – 1.51 | <0.001 | 1.22 | 1.07 – 1.41 | 0.004 |
| Deprivation [4] | 1.32 | 1.24 – 1.40 | <0.001 | 1.14 | 1.06 – 1.23 | <0.001 | 1.44 | 0.95 – 2.19 | 0.09 | 1.42 | 1.24 – 1.63 | <0.001 | 1.5 | 1.31 – 1.71 | <0.001 |
| Deprivation [5] Most deprived | 1.39 | 1.31 – 1.47 | <0.001 | 1.13 | 1.05 – 1.21 | 0.001 | 1.34 | 0.88 – 2.04 | 0.175 | 1.33 | 1.16 – 1.52 | <0.001 | 1.52 | 1.33 – 1.73 | <0.001 |
| Childhood Maltreatment | 1.25 | 1.16 – 1.35 | <0.001 | 1.2 | 1.09 – 1.33 | <0.001 | 1.29 | 0.78 – 2.13 | 0.322 | 1.32 | 1.12 – 1.56 | 0.001 | 1.37 | 1.19 – 1.59 | <0.001 |
| Self-harm | 1.7 | 1.43 – 2.04 | <0.001 | 1.36 | 1.08 – 1.71 | 0.008 | 2.35 | 1.03 – 5.41 | 0.044 | 1.84 | 1.29 – 2.63 | 0.001 | 1.74 | 1.27 – 2.38 | <0.001 |
| ADHD | 1.14 | 1.03 – 1.25 | 0.009 | 1.13 | 1.00 – 1.27 | 0.054 | 1.1 | 0.58 – 2.10 | 0.769 | 1.6 | 1.32 – 1.93 | <0.001 | 1.55 | 1.32 – 1.82 | <0.001 |
| ASD | 0.99 | 0.86 – 1.13 | 0.848 | 1.34 | 1.15 – 1.56 | <0.001 | 1.85 | 0.91 – 3.77 | 0.09 | 0.45 | 0.30 – 0.69 | <0.001 | 0.42 | 0.30 – 0.60 | <0.001 |
| Learning difficulties | 1.07 | 0.92 – 1.25 | 0.359 | 1.2 | 1.00 – 1.45 | 0.048 | 1.27 | 0.51 – 3.14 | 0.607 | 0.83 | 0.56 – 1.23 | 0.341 | 1.17 | 0.87 – 1.58 | 0.293 |
| Conduct disorder | 1.46 | 1.30 – 1.63 | <0.001 | 1.43 | 1.25 – 1.65 | <0.001 | 2.18 | 1.13 – 4.20 | 0.02 | 1.02 | 0.78 – 1.34 | 0.887 | 1.5 | 1.23 – 1.82 | <0.001 |
| Depression |  |  |  | 2.23 | 1.87 – 2.65 | <0.001 | 0.25 | 0.03 – 1.97 | 0.189 | 1.45 | 1.03 – 2.05 | 0.035 | 1.37 | 1.01 – 1.86 | 0.045 |
| Anxiety | 1.6 | 1.42 – 1.79 | <0.001 |  |  |  | 3.04 | 1.71 – 5.38 | <0.001 | 0.99 | 0.73 – 1.35 | 0.969 | 1 | 0.77 – 1.31 | 0.981 |
| Eating disorder | 1.4 | 1.18 – 1.65 | <0.001 | 1.4 | 1.13 – 1.74 | 0.002 |  |  |  | 0.67 | 0.39 – 1.14 | 0.141 | 1.25 | 0.87 – 1.81 | 0.226 |
| Alcohol misuse | 1.47 | 1.18 – 1.83 | 0.001 | 1.8 | 1.41 – 2.29 | <0.001 | 3.14 | 1.02 – 9.70 | 0.046 |  |  |  | 3.79 | 2.82 – 5.10 | <0.001 |
| Drugs misuse | 1.35 | 1.03 – 1.78 | 0.03 | 1.19 | 0.86 – 1.66 | 0.295 | 0.94 | 0.17 – 5.37 | 0.948 | 2.8 | 1.82 – 4.30 | <0.001 |  |  |  |
| Observations | 117211 | | | 117211 | | | 117211 | | | 117211 | | | 117211 | | |
| R^2^ Nagelkerke | 0.023 | | | 0.023 | | | 0.019 | | | 0.02 | | | 0.057 | | |
| * Accounting for EOTAS status (No/Yes), deprivation quintile, childhood maltreatment, self-harm, ND, and mental health conditions before the index date. IRR = incidence rate ratio. | | | | | | | | | | | | | | | |
