# Supplementary material for: Risk factors and trajectories for self-harm, neurodevelopmental disorders and mental health conditions in pupils in alternative education provision in Wales: population-based electronic cohort study
Source: BJPsych Open. 2025 Sep 11;11(5):e205. doi: 10.1192/bjo.2025.10827 (PMC12451533; doi:10.1192/bjo.2025.10827)
Supplement: Rouquette et al. supplementary material 3 — Rouquette et al. supplementary material [file S2056472425108272sup003.docx]

Supplementary table 4 - Logistic regression (robust standard error) computing the odds ratios of being in EOTAS provision separately for female and male*.

|  | **EOTAS Female** | | | **EOTAS Male** | | |
| --- | --- | --- | --- | --- | --- | --- |
| *Predictors* | *Odds Ratios* | *CI* | *p* | *Odds Ratios* | *CI* | *p* |
| Deprivation [2] | 1·34 | 1·13 – 1·59 | 0·001 | 1·51 | 1·32 – 1·73 | <0·001 |
| Deprivation [3] | 1·55 | 1·32 – 1·81 | <0·001 | 1·9 | 1·68 – 2·15 | <0·001 |
| Deprivation [4] | 2·18 | 1·88 – 2·53 | <0·001 | 2·63 | 2·34 – 2·96 | <0·001 |
| Deprivation [5] Most deprived | 2·68 | 2·33 – 3·10 | <0·001 | 3·96 | 3·54 – 4·43 | <0·001 |
| Childhood Maltreatment | 3·96 | 3·53 – 4·45 | <0·001 | 3·73 | 3·41 – 4·08 | <0·001 |
| Self-harm | 3·84 | 3·27 – 4·51 | <0·001 | 3·31 | 2·64 – 4·15 | <0·001 |
| ADHD | 3·08 | 2·33 – 4·08 | <0·001 | 4·16 | 3·73 – 4·64 | <0·001 |
| ASD | 3·87 | 2·72 – 5·51 | <0·001 | 2·09 | 1·77 – 2·45 | <0·001 |
| Learning difficulties | 2·86 | 2·04 – 4·00 | <0·001 | 1·55 | 1·25 – 1·92 | <0·001 |
| Conduct disorder | 5·35 | 4·27 – 6·71 | <0·001 | 3·51 | 3·04 – 4·04 | <0·001 |
| Depression | 3·09 | 2·65 – 3·60 | <0·001 | 3·97 | 3·29 – 4·79 | <0·001 |
| Anxiety | 3·71 | 3·23 – 4·28 | <0·001 | 3·02 | 2·60 – 3·50 | <0·001 |
| Eating disorder | 1·69 | 1·28 – 2·22 | <0·001 | 1·25 | 0·95 – 1·65 | 0·112 |
| Alcohol misuse | 3·14 | 2·37 – 4·16 | <0·001 | 3·27 | 2·43 – 4·42 | <0·001 |
| Drugs misuse | 2·22 | 1·60 – 3·09 | <0·001 | 2·83 | 1·96 – 4·08 | <0·001 |
| Observations | 112583 | | | 117211 | | |
| R^2^ Tjur | 0·083 | | | 0·089 | | |
| * Adjusted for deprivation, childhood maltreatment, self-harm, ND, and mental health conditions before the index date. | | | | | | |
