# Supplementary material for: Risk factors and trajectories for self-harm, neurodevelopmental disorders and mental health conditions in pupils in alternative education provision in Wales: population-based electronic cohort study
Source: BJPsych Open. 2025 Sep 11;11(5):e205. doi: 10.1192/bjo.2025.10827 (PMC12451533; doi:10.1192/bjo.2025.10827)
Supplement: Rouquette et al. supplementary material 4 — Rouquette et al. supplementary material [file S2056472425108272sup004.docx]

Supplementary Table 5 – Poisson model for self-harm, neurodevelopmental disorders, and mental health conditions*.

|  | **Self-harm** | | | **ADHD** | | | **ASD** | | | **Learning difficulties** | | | **Conduct disorder** | | |
| --- | --- | --- | --- | --- | --- | --- | --- | --- | --- | --- | --- | --- | --- | --- | --- |
| *Predictors* | *Incidence Rate Ratios* | *CI* | *p* | *Incidence Rate Ratios* | *CI* | *p* | *Incidence Rate Ratios* | *CI* | *p* | *Incidence Rate Ratios* | *CI* | *p* | *Incidence Rate Ratios* | *CI* | *p* |
| EOTAS [1] | 2·57 | 2·27 – 2·91 | **<0·001** | 6·35 | 4·50 – 8·96 | **<0·001** | 5·46 | 4·01 – 7·45 | **<0·001** | 4·68 | 3·03 – 7·24 | **<0·001** | 6·84 | 4·16 – 11·27 | **<0·001** |
| Sex [Male] | 0·53 | 0·50 – 0·55 | **<0·001** | 2·2 | 1·88 – 2·59 | **<0·001** | 1·79 | 1·55 – 2·07 | **<0·001** | 0·92 | 0·75 – 1·14 | 0·449 | 1·88 | 1·45 – 2·43 | **<0·001** |
| Deprivation [2] | 1·18 | 1·08 – 1·29 | **<0·001** | 0·89 | 0·70 – 1·14 | 0·351 | 1·07 | 0·86 – 1·34 | 0·551 | 1·44 | 1·01 – 2·05 | **0·044** | 1·14 | 0·75 – 1·74 | 0·525 |
| Deprivation [3] | 1·28 | 1·18 – 1·39 | **<0·001** | 1·05 | 0·84 – 1·31 | 0·656 | 1·2 | 0·98 – 1·48 | 0·082 | 1·37 | 0·97 – 1·93 | 0·076 | 1·38 | 0·93 – 2·03 | 0·106 |
| Deprivation [4] | 1·56 | 1·45 – 1·69 | **<0·001** | 1·22 | 0·99 – 1·50 | 0·064 | 1·3 | 1·07 – 1·59 | **0·01** | 1·52 | 1·09 – 2·11 | **0·014** | 1·24 | 0·84 – 1·81 | 0·277 |
| Deprivation [5] Most deprived | 1·64 | 1·52 – 1·77 | **<0·001** | 1·18 | 0·96 – 1·45 | 0·12 | 1·18 | 0·97 – 1·45 | 0·098 | 1·49 | 1·08 – 2·07 | **0·016** | 2·04 | 1·44 – 2·88 | **<0·001** |
| Childhood Maltreatment | 2·02 | 1·86 – 2·19 | **<0·001** | 1·89 | 1·52 – 2·36 | **<0·001** | 1·32 | 1·05 – 1·66 | **0·018** | 2·14 | 1·58 – 2·89 | **<0·001** | 1·78 | 1·27 – 2·48 | **0·001** |
| Self-harm | 0 | 0·00 – 0·00 | **<0·001** | 1·43 | 0·95 – 2·14 | 0·083 | 0·74 | 0·47 – 1·16 | 0·193 | 1·14 | 0·63 – 2·09 | 0·662 | 1·69 | 1·00 – 2·86 | **0·049** |
| ADHD | 1·61 | 1·42 – 1·84 | **<0·001** | 0 | 0·00 – 0·00 | **<0·001** | 3·11 | 2·44 – 3·97 | **<0·001** | 3·52 | 2·35 – 5·26 | **<0·001** | 2·74 | 1·88 – 3·99 | **<0·001** |
| ASD | 0·93 | 0·75 – 1·14 | 0·482 | 2·15 | 1·49 – 3·11 | **<0·001** | 0 | 0·00 – 0·00 | **<0·001** | 3·96 | 2·61 – 6·03 | **<0·001** | 0·86 | 0·44 – 1·69 | 0·665 |
| Learning difficulties | 1·07 | 0·86 – 1·33 | 0·551 | 1·86 | 1·21 – 2·86 | **0·004** | 3·89 | 2·90 – 5·22 | **<0·001** | 0 | 0·00 – 0·00 | **<0·001** | 2 | 1·10 – 3·65 | **0·023** |
| Conduct disorder | 1·5 | 1·29 – 1·75 | **<0·001** | 1·79 | 1·27 – 2·53 | **0·001** | 2·01 | 1·51 – 2·68 | **<0·001** | 1·66 | 1·07 – 2·59 | **0·025** | 0 | 0·00 – 0·00 | **<0·001** |
| Depression | 2·38 | 2·10 – 2·70 | **<0·001** | 1·6 | 1·12 – 2·30 | **0·011** | 2·53 | 1·86 – 3·45 | **<0·001** | 0·74 | 0·35 – 1·54 | 0·418 | 1·49 | 0·86 – 2·60 | 0·157 |
| Anxiety | 1·32 | 1·17 – 1·50 | **<0·001** | 1·25 | 0·89 – 1·75 | 0·201 | 2·62 | 2·04 – 3·36 | **<0·001** | 1 | 0·59 – 1·70 | 0·993 | 1·4 | 0·84 – 2·33 | 0·203 |
| Eating disorder | 1·36 | 1·12 – 1·66 | **0·002** | 0·95 | 0·51 – 1·78 | 0·884 | 1·56 | 0·99 – 2·47 | 0·057 | 1·68 | 0·86 – 3·30 | 0·131 | 1·07 | 0·44 – 2·58 | 0·879 |
| Alcohol misuse | 2·6 | 2·16 – 3·13 | **<0·001** | 1·26 | 0·71 – 2·23 | 0·423 | 0·38 | 0·14 – 1·02 | 0·055 | 0·62 | 0·20 – 1·93 | 0·41 | 1·32 | 0·61 – 2·85 | 0·478 |
| Drugs misuse | 2·03 | 1·50 – 2·74 | **<0·001** | 0·66 | 0·29 – 1·47 | 0·305 | 0·73 | 0·30 – 1·77 | 0·489 | 0·78 | 0·25 – 2·40 | 0·661 | 1·38 | 0·65 – 2·95 | 0·399 |
| EOTAS [1] * Sex [Male] | 1·25 | 1·07 – 1·46 | **0·005** | 1·03 | 0·71 – 1·49 | 0·872 | 0·62 | 0·44 – 0·89 | **0·008** | 0·79 | 0·47 – 1·31 | 0·357 | 1 | 0·58 – 1·71 | 0·987 |
| Observations | 229794 | | | 229794 | | | 229794 | | | 229794 | | | 229794 | | |
| R^2^ Nagelkerke | 0·057 | | | 0·071 | | | 0·07 | | | 0·068 | | | 0·084 | | |
|  | **Depression** | | | **Anxiety** | | | **Eating disorder** | | | **Alcohol misuse** | | | **Drugs misuse** | | |
| *Predictors* | *Incidence Rate Ratios* | *CI* | *p* | *Incidence Rate Ratios* | *CI* | *p* | *Incidence Rate Ratios* | *CI* | *p* | *Incidence Rate Ratios* | *CI* | *p* | *Incidence Rate Ratios* | *CI* | *p* |
| EOTAS [1] | 1·8 | 1·69 – 1·93 | **<0·001** | 1·6 | 1·47 – 1·74 | **<0·001** | 1·16 | 0·84 – 1·59 | 0·372 | 2·31 | 1·92 – 2·78 | **<0·001** | 4·17 | 3·55 – 4·90 | **<0·001** |
| Sex [Male] | 0·5 | 0·49 – 0·51 | **<0·001** | 0·42 | 0·41 – 0·43 | **<0·001** | 0·18 | 0·15 – 0·21 | **<0·001** | 1·02 | 0·95 – 1·08 | 0·593 | 1·46 | 1·36 – 1·56 | **<0·001** |
| Deprivation [2] | 1·08 | 1·04 – 1·13 | **<0·001** | 0·99 | 0·95 – 1·04 | 0·796 | 0·96 | 0·81 – 1·14 | 0·637 | 1·12 | 1·01 – 1·25 | **0·04** | 1·11 | 0·99 – 1·25 | 0·07 |
| Deprivation [3] | 1·15 | 1·11 – 1·20 | **<0·001** | 1·05 | 1·00 – 1·09 | **0·036** | 0·91 | 0·77 – 1·07 | 0·254 | 1·28 | 1·16 – 1·42 | **<0·001** | 1·2 | 1·07 – 1·33 | **0·001** |
| Deprivation [4] | 1·32 | 1·28 – 1·37 | **<0·001** | 1·15 | 1·10 – 1·20 | **<0·001** | 0·8 | 0·68 – 0·94 | **0·008** | 1·32 | 1·19 – 1·46 | **<0·001** | 1·5 | 1·35 – 1·66 | **<0·001** |
| Deprivation [5] Most deprived | 1·42 | 1·37 – 1·47 | **<0·001** | 1·15 | 1·10 – 1·20 | **<0·001** | 0·78 | 0·67 – 0·92 | **0·003** | 1·33 | 1·20 – 1·46 | **<0·001** | 1·54 | 1·39 – 1·70 | **<0·001** |
| Childhood Maltreatment | 1·42 | 1·36 – 1·49 | **<0·001** | 1·28 | 1·21 – 1·35 | **<0·001** | 1·63 | 1·32 – 2·03 | **<0·001** | 1·66 | 1·48 – 1·87 | **<0·001** | 1·73 | 1·55 – 1·92 | **<0·001** |
| Self-harm | 2·06 | 1·92 – 2·22 | **<0·001** | 1·41 | 1·29 – 1·54 | **<0·001** | 2·07 | 1·56 – 2·75 | **<0·001** | 2·1 | 1·74 – 2·54 | **<0·001** | 2·48 | 2·09 – 2·93 | **<0·001** |
| ADHD | 1·13 | 1·04 – 1·22 | **0·003** | 1·23 | 1·12 – 1·35 | **<0·001** | 1·28 | 0·84 – 1·94 | 0·258 | 1·6 | 1·35 – 1·89 | **<0·001** | 1·56 | 1·35 – 1·81 | **<0·001** |
| ASD | 0·98 | 0·88 – 1·10 | 0·782 | 1·34 | 1·18 – 1·52 | **<0·001** | 1·53 | 0·91 – 2·57 | 0·109 | 0·47 | 0·32 – 0·67 | **<0·001** | 0·41 | 0·30 – 0·56 | **<0·001** |
| Learning difficulties | 1·05 | 0·93 – 1·18 | 0·411 | 1·07 | 0·92 – 1·23 | 0·382 | 1·22 | 0·70 – 2·13 | 0·486 | 0·88 | 0·64 – 1·21 | 0·426 | 1·06 | 0·81 – 1·38 | 0·684 |
| Conduct disorder | 1·5 | 1·39 – 1·63 | **<0·001** | 1·36 | 1·23 – 1·51 | **<0·001** | 1·36 | 0·89 – 2·09 | 0·16 | 1·1 | 0·89 – 1·37 | 0·389 | 1·55 | 1·31 – 1·83 | **<0·001** |
| Depression | 0 | 0·00 – 0·00 | **<0·001** | 2·07 | 1·92 – 2·23 | **<0·001** | 1·38 | 1·03 – 1·87 | **0·033** | 1·64 | 1·36 – 1·97 | **<0·001** | 1·82 | 1·53 – 2·16 | **<0·001** |
| Anxiety | 1·6 | 1·51 – 1·70 | **<0·001** | 0 | 0·00 – 0·00 | **<0·001** | 1·84 | 1·44 – 2·35 | **<0·001** | 1 | 0·82 – 1·21 | 0·989 | 1 | 0·83 – 1·20 | 0·979 |
| Eating disorder | 1·38 | 1·25 – 1·52 | **<0·001** | 1·27 | 1·13 – 1·43 | **<0·001** | 0 | 0·00 – 0·00 | **<0·001** | 1·04 | 0·78 – 1·39 | 0·767 | 1·14 | 0·87 – 1·49 | 0·342 |
| Alcohol misuse | 1·39 | 1·24 – 1·56 | **<0·001** | 1·39 | 1·22 – 1·58 | **<0·001** | 1·38 | 0·86 – 2·21 | 0·188 | 0 | 0·00 – 0·00 | **<0·001** | 3·05 | 2·46 – 3·77 | **<0·001** |
| Drugs misuse | 1·2 | 1·03 – 1·40 | **0·019** | 1 | 0·84 – 1·20 | 0·999 | 0·6 | 0·28 – 1·31 | 0·2 | 1·98 | 1·45 – 2·70 | **<0·001** | 0 | 0·00 – 0·00 | **<0·001** |
| EOTAS [1] * Sex [Male] | 0·97 | 0·88 – 1·06 | 0·459 | 1·23 | 1·10 – 1·38 | **<0·001** | 1·77 | 1·05 – 2·98 | **0·031** | 1 | 0·80 – 1·24 | 0·969 | 0·95 | 0·79 – 1·14 | 0·592 |
| Observations | 229794 | | | 229794 | | | 229794 | | | 229794 | | | 229794 | | |
| R^2^ Nagelkerke | 0·067 | | | 0·067 | | | 0·061 | | | 0·022 | | | 0·065 | | |
| * Models adjusted for EOTAS status (No/Yes), Sex (Female/Male) deprivation quintile, childhood maltreatment, self-harm, ND, and mental health conditions before the index date· | | | | | | | | | | | | | | | |
